# Retraction: Suppression of microRNA-205-5p in human mesenchymal stem cells improves their therapeutic potential in treating diabetic foot disease

**DOI:** 10.18632/oncotarget.28796

**Published:** 2025-12-31

**Authors:** Lingyan Zhu, Gongxian Wang, Shane Fischbach, Xiangwei Xiao

**Affiliations:** ^1^Department of Endocrinology, The First Affiliated Hospital of Nanchang University, Nanchang 330006, China; ^2^Department of Urology, The First Affiliated Hospital of Nanchang University, Nanchang 330006, China; ^3^Division of Pediatric Surgery, Department of Surgery, Children’s Hospital of Pittsburgh, University of Pittsburgh School of Medicine, Pittsburgh, PA15224, USA; ^*^These authors contributed equally to this work

**This article has been retracted:** Oncotarget has completed its investigation of this paper. An image forensic analysis revealed that the von Kossa image in Figure 4B overlaps with an image in Figure 1D of an earlier paper [1]. Furthermore, part of the same von Kossa staining image was reproduced in at least four subsequent publications [2–5], where also the part of the image in Figure 4C “Differentiation of as-miR-205-5p-MSCs into adipocytes by Oil red O staining” was reproduced in Figure 1E of [2], Figure 3E of [3], Figure 1B of [4], and Figure 1E of [5]. Additionally, western blot in Figure 1B contains a duplicate image from Figure 3D of another earlier paper [6], which shares two authors (Shane Fischbach and Xiangwei Xiao) with the retracted article. The documentation provided by corresponding author Xiangwei Xiao regarding the von Kossa staining and western blots did not adequately address the concerns raised. Therefore, the editorial decision was made to retract this paper.

Original article: Oncotarget. 2017; 8:52294–52303. 52294-52303. https://doi.org/10.18632/oncotarget.17012
